# Knowledge and Awareness of Reproductive Rights Among Women With Schizophrenia and Bipolar Affective Disorder

**DOI:** 10.7759/cureus.106264

**Published:** 2026-04-01

**Authors:** Gobinda Majhi, Ponnuchamy Lingam, Vranda M N, Shreedevi A U

**Affiliations:** 1 Department of Psychiatric Social Work, National Institute of Mental Health and Neurosciences, Bengaluru, IND

**Keywords:** knowledge and awareness, mental illness, reproductive rights, wellbeing, women

## Abstract

Background: Female psychiatric patients face challenges in reproductive health due to a lack of knowledge and awareness about reproductive rights and limited access to healthcare facilities. Our primary objectives were to explore the level of knowledge and awareness, independence, autonomy, and decision-making power concerning reproductive rights among women with schizophrenia and bipolar affective disorder (BPAD).

Materials and methods: The study employed a cross-sectional descriptive design and included 150 women with schizophrenia and BPAD using a purposive sampling technique in a tertiary care mental health setting in India and diagnosed according to ICD-10. The Clinical Global Impression (CGI, Improvement) and a predesigned questionnaire about fundamental rights, knowledge and awareness, liberty and freedom, and autonomy and decision-making of women with mental illness were used for data collection.

Results: The majority of 120 (80.0%) of the respondents, irrespective of schizophrenia and BPAD, opined that females should be provided with proper education and awareness to help them become emotionally and physical easy. A total of 120 (80%) feel that girls should be educated in maintaining the highest level of hygiene during periods.

Conclusion: Women with schizophrenia and BPAD should be offered comprehensive counseling and awareness related to reproductive health, including accessing reproductive health services.

## Introduction

The World Health Organization declares reproductive rights as one of the fundamental human rights. These rights ensure that individuals have the autonomy to make informed choices about their bodies, including decisions about having children, free from coercion and discrimination. These rights are among the critical public health concerns for women with mental illness, where socio-cultural, religious, and economic factors determine the health outcome. However, many women with mentally ill persons are deprived of their rights by forced sterilization without proper consent. Decisions about pregnancy are taken by family or caregivers without the woman's consent, that is, being confined at home or in institutions against their will. In recent years, an exponential increase in violations of the rights of persons with mental illness has been reported worldwide [1]. Various studies also reported that women from low socio-economic status with mental illness are particularly more vulnerable to abuse and exploitation and face barriers to reproductive health [2-4]. For instance, low rates of using contraceptive methods [5], decreased options for marriage [6], risky sexual practices [7,5], increased rates of unplanned pregnancies [5], and fewer opportunities to make informed decisions [8] are major challenges for women with psychiatric illnesses. Many studies focused on reproductive health, where issues and problems centered around transmission of diseases, and risky sexual behaviors [9,10]. Especially in low- and middle-income countries, unintended pregnancies have resulted in many unsafe abortions and caused maternal death [11,12]. Unintended pregnancy could be the result of many factors, such as not being a user of contraception, incorrect use of contraceptive, education, and women's autonomy [13]. Therefore, there is a need to address the issues related to reproductive rights, knowledge, and autonomy. Limited autonomy and decision-making power can highly influence the individual's informed choice regarding contraception, pregnancy, and healthcare access. Hence, the aim of this study was to understand the level of knowledge and awareness regarding reproductive rights, decision-making, and autonomy of women with schizophrenia and bipolar affective disorder (BPAD).

## Materials and methods

Study design

The study employed a cross-sectional descriptive design.

Study population****

The study population included all women with schizophrenia and BPAD attending the Outpatient Department (OPD) of the National Institute of Mental Health and Neuro Sciences (NIMHANS), Bengaluru District, Karnataka State, India.

Sample size

We approached 300 women with schizophrenia and BPAD, out of which 150 and 140 were screened, respectively, among which 83 (schizophrenia group) and 67 (BPAD group) met the eligibility criteria. Hence, the total sample for this study was 150. The sample size was determined based on feasibility, considering the study duration, available resources, and patient attendance at the study setting.

Sampling strategy

The purposive sampling technique was used for the study. This method allowed us to conduct a study in the OPD to capture the participants' direct experience of the phenomenon under study within the stipulated timeframe.

Study setting

The study setting was women with schizophrenia and BPAD receiving treatment from the OPD of NIMHANS.

Study duration 

The data collection was conducted between August 2023 and May 2024.

Study measures

A socio-demographic data sheet was used to record personal and clinical characteristics of the participants. The Clinical Global Impression (CGI) [14] was used for noting the symptom severity and improvement. CGI is a 7-point scale that requires the clinician to assess how much the patient's illness has improved or worsened relative to a baseline and is rated as follows: 1, very much improved; 2, much improved; 3, minimally improved; 4, no change; 5, minimally worse; 6, much worse; or 7, very much worse. The CGI is available in open access, public domain. Hence, it does not require permission to administer it. In addition, a predesigned questionnaire, which consisted of 27 questions categorized under four sub-domains include fundamental rights (3 items), knowledge and awareness (16 items), liberty and freedom (3 items), and women's autonomy and decision-making (5 items) to understand knowledge and awareness about the reproductive rights of women with mental illness, was used for data collection. It was validated by five experts. All the experts are affiliated with the same institution but from different disciplines, for example, two professors of psychiatric social work, two professors of psychiatry, and one professor of clinical psychology. Selection was based on a minimum of 10 years' experience in teaching and research in psychiatric settings. They were approached for the questionnaire validation, they accepted and validated it, and none of them are part of the authors of this article. 

Eligibility criteria

The study had the following inclusion criteria: All the subjects diagnosed with schizophrenia and BPAD according to the International Classification of Diseases, 10th Revision (ICD-10), the participants should be aged between 18 and 40 years, and they should be able to speak any one of the following languages: Kannada, Hindi, English, or Tamil. The exclusion criteria were those persons having acute symptoms and not providing written informed consent. The exclusion criteria were limited to factors that could directly impair participants' ability to comprehend, respond reliably, or provide informed consent, thereby ensuring data validity.

Ethics statement

The institute's ethical clearance was obtained from the Institutional Ethics Committee (IEC) of NIMHANS, before commencing the study (No. NIMHANS/EC (BEH, SC.DIV) Meeting/2023 Date: 18.08.2023).

Study procedure

After getting ethical permission, all participants who met the inclusion criteria were selected during follow-up visits to the OPD of NIMHANS. They were informed of the study's purpose. Those who gave informed consent were administered a demographic data sheet specifically designed for the study, and a CGI and content-validated questionnaire was administered to the selected sample. The data were collected by the research assistant appointed for this purpose. We have ensured the privacy, comfort, and confidentiality of the participants. The data collection was done using the pen and paper method during the normal course of outpatient services at the convenience of the participants. If the participants were illiterate, then the questionnaire was read out by the research assistant appointed for this purpose.

Statistical analysis 

Statistical Product and Service Solutions (SPSS, version 26.0; IBM SPSS Statistics for Windows, Armonk, NY) was utilized for the data analysis. Normality tests, including the Shapiro-Wilk and Kolmogorov-Smirnov tests, were performed; they indicated that the data do not follow a normal distribution, allowing non-parametric tests. Descriptive statistics include frequency, percentage, mean, and standard deviation. A Mann-Whitney test was used to assess the statistical significance of the mean differences between diagnosis and demographic and clinical variables, age, age at onset, and duration of illness. Further, the chi-square test was used to determine the statistical differences in patients' diagnosis and fundamental rights, knowledge and awareness, liberty and freedom, and autonomy and decision-making. The significance level was set at p < 0.05 to describe the statistics.

## Results

Table 1 shows the demographic distribution of the respondents. The participants' marital distribution shows that the majority (44 (53%) in schizophrenia and 38 (56%) in BPAD) were single. In the religion distribution, 71 (85.5%) with schizophrenia and 62 (92.5%) with BPAD were Hindu. In domicile distribution, the majority (48 (57.8%) with schizophrenia and 38 (56.7%) with BPAD) were from a rural background. In the occupation distribution, 31 (37.3%) with schizophrenia and 19 (28.4%) with BPAD were housewives. Regarding the preponderance of the subject's educational background, 24 (28.9%) with schizophrenia and 19 (28.4%) with BPAD studied up to secondary levels. The income distribution shows that the majority in the schizophrenia group belongs to middle socio-economic status (44, 53%), whereas in BPAD, the majority (46, 68.6%) belongs to lower socio-economic status. The family type distribution shows that 79 (95.2%) with schizophrenia and 64 (96.5%) with BPAD were from a nuclear family. The course of illness distribution shows that the majority (60, 72.3%) with schizophrenia and (47, 70.1%) with BPAD had progressive illnesses. In the majority of the cases (78 (94.0%) with schizophrenia and 65 (97.0%) with BPAD), the precipitating factors were unknown. The CGI score indicates that the majority (54 (64.1%) with schizophrenia and 40 (59.7%) with BPAD) were moderately ill, respectively.

**Table 1:** Demographic characteristics of the participants (N=150) BPAD: bipolar affective disorder; CGI: Clinical Global Impression

Variables		Schizophrenia, N (%)	BPAD, N (%)	χ^2^	df	p-value
Marital status	Single	44 (53.0)	38 (56.7)	0.219	2	0.896
	Married	34 (41.0)	25 (37.3)			
	Others	5 (6.0)	4 (6.0)			
Religion	Hindu	71 (85.5)	62 (92.5)	3.947	3	0.267
	Muslim	6 (7.2)	2 (3.0)			
	Christian	6 (7.2)	2 (3.0)			
	Others	0 (0.0)	1 (1.5)			
Domicile	Rural	48 (57.8)	38 (56.7)	0.019	1	0.891
	Urban	35 (42.2)	29 (43.3)			
Occupation	House wife	31 (37.3)	19 (28.4)	3.592	3	0.309
	teacher	2 (2.4)	4 (6.0)			
	Professional	1 (1,2)	3 (4.5)			
	Others	49 (59.0)	41 (61.2)			
Educational status	Illiterate	17 (20.5)	7 (10.4)	4.663	3	0.198
	Primary	20 (24.1)	14 (20.4)			
	secondary	24 (28.9)	19 (28.4)			
	Others	22 (26.5)	41 (61.2)			
Income	Low-SES	37 (44.6)	46 (46.3)	0.106	2	0.948
	Middle -SES	44 (53.0)	34 (50.7)			
	High-SES	2 (2.4)	3 (3.0)			
Family type	Nuclear	79 (95.2)	64 (96.5)	0.010	1	0.921
	Joint	4 (4.8)	3 (3.0)			
Course of illness	Continuous	7 (8.4)	10 (14.9)	1.807	1	0.405
	Progress	60 (72.3)	47 (70.1)			
	Static	16 (19.3)	10 (14.9)			
Precipitating factors	Known precipitating factors	5 (6.0)	2 (3.0)	0.770	1	0.380
	Unknown precipitating factors	78 (94.0)	65 (97.0)			
Severity of illness - CGI	Normal, not at all ill	1 (1.2)	0 (00)			
	Borderline mentally ill	11 (13.3)	6 (9.0)			
	Mildly ill	17 (20.5)	21 (31.3)	3.308	3	0.347
	Moderately ill	54 (64.1)	40 (59.7)			

Table 2 shows the clinical characteristics of the participants. The total number of participants was 150, among whom 83 (55.33%) had schizophrenia and 67 (44.66%) had BPAD. The mean age of the respondents having schizophrenia is 28.54 (SD=6.50), whereas the mean age of respondents having BPAD is 28.33 (SD=5.70). On the other hand, the mean age of the onset of participants with schizophrenia and BPAD was 23.47 (SD=5.62) and 22.48 (SD=5.75), respectively. The duration of illness was higher in the BPAD group, with a mean of 6.16 (SD=5.35), than in the schizophrenia group (mean=5.72; SD=4.40). 

**Table 2:** Clinical characteristics of the participants (N=150) BPAD: bipolar affective disorder

Variable	Group	N	Mean	SD
Age	Schizophrenia	83	28.54	6.50
	BPAD	67	28.33	5.70
Age of onset	Schizophrenia	83	23.47	5.62
	BPAD	67	22.48	5.75
Duration of illness	Schizophrenia	83	5.72	4.40
	BPAD	67	6.16	5.35

Table 3 shows the knowledge and awareness of the respondents on reproductive rights. Apart from their spouses, 11 (7.3%) had other sexual partners in the last 12 months. Regarding the knowledge and awareness of reproductive rights of the respondents, 67 (44.7%) respondents believes that reproductive rights are their fundamental rights; 74 (49.3%) respondents feel that unjustified medication can harm reproductive health; 90 (60%) participants feel that medication should be provided to meet the best health needs of a patient; 67 (44.7%) feel that sterilization should not be carried out as a treatment for mental illness; 75 (50.0%) were aware that, during illness period, conceiving or giving birth could impact mental health; 44 (29.3%) do not have knowledge on how to prevent unwanted pregnancy; and 50 (33.3%) were not aware about the different contraceptive methods available in India. Fifty-three (35%) respondents are not aware of injectables as a type of contraceptive, and 102 (68.0%) accepted that it is healthy to get a period at a young age. Moreover, 80.0% of respondents feel that girls should be provided with proper education and awareness to help them become emotionally and physically ready. One hundred twenty (78.0%) respondents feel that girls should be educated in maintaining the highest level of hygiene during periods, 113 (75.3%) feel that the lack of access to high-quality hygiene products is a major impediment, 101 (67.3%) agree that women and girls do not have access to adequate sanitation facilities, 104 (69.3%) feel that restrictions imposed on girls during menstruation are the right move, 122 (81.3%) feel that girls should receive comprehensive counselling on menstruation and other associated issue, 69 (46%) have not ever planned to have child birth, 25 (24.7%) had ever miscarriage or aborted or stillbirth child, 14 (9%) accepted that they have been ever controlled sexually, and 60 (40%) expressed a preference for having a baby now. In addition, 28 (19%) participants expressed that they were dependent upon the partner and consider them in the decision to have a child, 65 (43.3%) had plan to have a child in the near future, 22 (14.7%) participants' marriage was not solemnized on their own choice, and 37 (24.7%) had not hear about family planning method.

**Table 3:** Respondents' knowledge and awareness of reproductive rights (N=150) BPAD: bipolar affective disorder

Sl. No	Questions		Schizophrenia, N (%)	BPAD, N (%)	X^2^	DF	P-value
Awareness about the fundamental rights							
1	Are reproductive rights fundamental rights?	Yes	33 (39.8)	34 (50.7)	3.698	2	0.296
		No	25 (30.1)	21 (31.3)			
		Don’t know	25 (30.1)	12 (17.9)			
2	Apart from your husband, have you had any other sexual partners in the last 12 months?	Yes	7 (8.4)	4 (6.0)	0.332	2	0.847
		No	42 (50.6)	35 (52.2)			
		Don’t know	34 (41.0)	28 (41.8)			
3	Do you feel restrictions imposed on girls during menstruation is right move?	Yes	61 (73.5)	43 (64.2)	1.514	2	0.469
		No	12 (14.5)	13 (19.4)			
		Don’t know	10 (12.0)	11 (16.4)			
Knowledge and awareness about reproductive health							
4	Do you feel that unjustified medication can harm you and your reproductive health?	Yes	39 (47.0)	35 (52.2)	2.688	2	0.442
		No	22 (26.5)	21 (31.3)			
		Don’t know	22 (26.5)	11 (16.4)			
5	Do you feel medication should be provided to meet the best health needs of the patient?	Yes	51 (61.4)	39 (58.2)	4.031	2	0.258
		No	13 (15.7)	18 (26.9)			
		Don’t know	19 (22.9)	10 (14.9)			
6	Do you feel that sterilization should not be carried out as a treatment for mental illness?	Yes	35 (42.2)	32 (47.8)	2.175	2	0.537
		No	23 (27.7)	21 (31.3)			
		Don’t know	25 (30.1)	14 (20.9)			
7	Are you aware that during an illness period, conceiving or giving birth could impact your mental health?	Yes	37 (44.6)	38 (56.7)	4.628	2	0.201
		No	19 (22.9)	17 (25,4)			
		Don’t know	27 (32.5)	12 (17.9)			
8	Do you have knowledge of how to prevent unwanted pregnancy?	Yes	38 (45.8)	31 (46.3)	0.066	2	0.968
		No	25 (31.1)	19 (28.4)			
		Don’t know	20 (24.1)	17 (25.4)			
9	Do you know the source of information regarding puberty and menstruation?	Yes	79 (95.2)	63 (94.0)	4.952	2	0.084
		No	3 (3.6)	0 (00)			
		Don’t know	1 (1.2)	4 (6.0)			
10	Before reaching the age of menarche, did you receive any information about menstruation?	Yes	77 (92.8)	57 (85.1)	2.912	2	0.233
		No	3 (3.6)	7 (10.4)			
		Don’t know	3 (3.6)	3 (4.5)			
11	Are you aware of the different contraceptive methods available in India?	Yes	41 (49.4)	30 (44.8)	0.392	2	0.804
		No	26 (31.3)	24 (35.8)			
		Don’t know	16 (19.3)	13 (19.4)			
12	Do you know about injectables as a type of contraceptive?	Yes	40 (48.2)	25 (37.3)	1.921	2	0.383
		No	26 (31.3)	27 (40.0)			
		Don’t know	17 (20.5)	15 (22.4)			
13	Do you know any source of information on contraceptives?	Yes	77 (92.8)	62 (92.5)	2.406	2	0.314
		No	4 (4.8)	1 (1.5)			
		Don’t know	2 (2.4)	4 (6.0)			
14	Is it healthy to get your period at a young age?	Yes	59 (71.1)	43 (64.2)	1.172	2	0.557
		No	16 (19.3)	14 (20.9)			
		Don’t know	8 (9.6)	10 (14.9)			
15	Do you feel girls should be provided with proper education and awareness to help them become emotionally and physically ready?	Yes	70 (84.3)	50 (74.6)	3.806	2	0.149
		No	5 (6.0)	3 (4.5)			
		Don’t know	8 (9.6)	14 (20.9)			
16	Do you feel girls should be educated in maintaining the highest level of hygiene during their periods?	Yes	67 (80.7)	50 (74.6)	1.540	2	0.463
		No	8 (9.6)	6 (9.0)			
		Don’t know	8 (9.6)	11 (16.4)			
17	Do you ever feel lack of access to high-quality hygiene products is a major impediment?	Yes	66 (79.5)	47 (70.1)	3.861	2	0.145
		No	10 (12.0)	7 (10.4)			
		Don’t know	7 (8.4)	13 (19.4)			
18	Do you agree that women and girls do not have access to adequate sanitation facilities?	Yes	60 (72.3)	41 (61.2)	4.024	2	0.134
		No	16 (19.3)	13 (19.4)			
		Don’t know	7 (8.4)	13 (19.4)			
19	Do you feel girls should receive comprehensive counselling on menstruation and other associated issue?	Yes	69 (83.1)	53 (79.1)	0.541	2	0.763
		No	7 (8.4)	6 (9.0)			
		Don’t know	7 (8.4)	8 (11.9)			
Exercise of liberty and freedom of choice in reproduction							
20	Have you ever planned to have a child?	Yes	30 (36.1)	26 (38.8)	0.600	2	0.741
		No	36 (43.4)	25 (37.3)			
		Don’t know	17 (20.5)	16 (23.9)			
21	Have you ever had a miscarriage?	Yes	23 (27.7)	14 (20.9)	2.320	2	0.314
		No	47 (56.6)	46 (68.7)			
		Don’t know	13 (15.7)	7 (10.4)			
22	Has your partner ever controlled you sexually?	Yes	8 (9.6)	6 (9,0)	0.058	2	0.967
		No	21 (25.3)	18 (26.9)			
		Don’t know	54 (65.1)	43 (64.2)			
Women’s autonomy in reproductive health decision-making							
23	Do you feel that you should have a baby now?	Yes	34 (41.0)	26 (38.8)	0.122	2	0.955
		No	31 (37.3)	25 (37.3)			
		Don’t know	18 (21.7)	16 (239)			
24	Do you solely make the decision to have a child?	Yes	42 (50.6)	33 (49.3)	0.421	2	0.810
		No	14 (16.9)	14 (20.9)			
		Don’t know	27 (32.5)	20 (29.9)			
25	Was your marriage solemnized by your own choice?	Yes	42 (50.6)	35 (52.2)	3.005	2	0.223
		No	9 (10.8)	13 (19.4)			
		Don’t know	32 (38.6)	19 (28.4)			
26	Do you intend to have a child in the near future?	Yes	36 (43.4)	29 (43.3)	0.086	2	0958
		No	22 (26.5)	19 (28.4)			
		Don’t know	25 (30.1)	19 (28.4)			
27	Have you ever heard about family planning methods?	Yes	49 (59.0)	31 (46.3)	4.476	2	0.107
		No	15 (18.1)	22 (31.8)			
		Don’t know	19 (22.9)	14 (20.9)			

## Discussion

The current study aimed to understand the level of knowledge and awareness regarding reproductive rights among women with schizophrenia and BPAD. A total of 150 participants were selected using a purposive sampling technique. Both women with schizophrenia and BPAD patients were recruited for the study.

Awareness about the fundamental rights

According to the Indian Constitution, under Article 21, the right to life guarantees personal liberty and reproductive choices too. Conversely, the present study findings show that 30.7% of respondents do not know whether reproductive rights are fundamental rights. Despite India having passed the Medical Termination of Pregnancy Act, 1971 (later amended in 2021) and prepared a family planning policy framework to ensure access to contraception and abortion, women and girls still face significant obstacles to fully exercising their reproductive rights [15-17]. Further, women with severe mental illnesses are at risk for sexual health-related challenges, as the present study found that 7.2% participants reported that they had unprotected sex or high-risk sexual behaviour with partners other than their spouse. This is in line with the research conducted by Coverdale et al. [18]. It suggests that persons with schizophrenia and severe mental illness have had more sexual partners, non-consensual sex, and HIV-risk behavior than those without mental illness. Poor awareness about sexual rights and health practices is also reflected in 68.85% respondents endorsing that restrictions imposed on girls during menstruation are the right move [19]. Studies conducted by Guterman et al. and Gottlieb support the present study's finding that different religions have enduring beliefs and taboos that restrict women from entering holy places during menstruation, as it is often perceived to render the space impure [19,20]. Additionally, 68.85% of the respondents in our study expressed that restrictions imposed on girls during menstruation are the right move. In response to the above findings, the following description was included as supporting evidence. These findings have clinical implications in mental health settings. Individuals with limited understanding may not seek services. Hence, integrating with a psychoeducation programme in the mental health service programme would improve the awareness and autonomy among the women. Further, incorporating rights-based counseling will empower patients to understand their reproductive choices better and exercise them.

Knowledge and awareness about reproductive health

Our study findings show that 28.7% of respondents feel that unjustified medication may not harm reproductive health. The present study's findings were contrasted with the previous studies conducted by Thompson et al. and Al Jishi et al. [21,22]. The findings revealed that 25.2% of respondents lack awareness about preventing unwanted pregnancy and childbirth and mental health-related issues, including knowledge of contraceptive methods. The present study concurs with the previous study, which indicates that limited access to reliable reproductive health information contributes to high rates of unintended pregnancy and unsafe abortion among adolescents. It contradicts the previous study findings, which suggest that conceiving or giving birth would impact mental health [23,24]. In this study, 94.6% of respondents had a known source of information about puberty and menstruation; in the majority of the cases, it is the mother [25]. Our study findings indicate that 79.45% of respondents feel that girls should be provided with proper education and awareness to help them become emotionally and physically ready, which is similar to other studies [26,27]. The present study's findings demonstrate that 79.45% of respondents feel that girls should be educated to maintain the highest level of hygiene during periods. Similar findings were reported by Das et al. [28]. The lack of awareness and restriction imposed on girls often reflects broader gaps in the community about reproductive rights. Further, socio-economic factors may influence education, reduce access to healthcare facilities, and be shaped by religious and cultural norms, which may, in turn, influence decision-making and control over their own bodies.

Our study findings show that 74.8% of respondents perceive a lack of access to high-quality hygiene products as a significant barrier demonstrated multiple underlying challenges. A lack of access may not be the only problem; many factors may influence it, such as irregular supply of sanitary products, unaffordability, limited markets, and distance to the place [29-33]. Our study findings indicate that 66.75% respondents agree that women and girls do not have access to adequate sanitation facilities, such as infrastructure and services that enable individuals - especially women and girls - to maintain hygiene safely and with dignity, particularly during menstruation, pregnancy, childbirth, and postpartum periods. The present study findings were supported by previous studies conducted previous studies [33-35]. The study findings show that 81.1% of respondents feel that girls should receive comprehensive counselling on menstruation and other associated issues, such as hygiene, physical symptoms, emotional well-being, and social challenges. The present study was supported by a previous study conducted by Burke [36]. Interestingly, our findings also indicate that 69.3% of respondents considered the restriction imposed during menstruation to be an appropriate move, which reflects the sociocultural norms of the Indian society. It is very significant from a clinical point of view, as strong beliefs may influence health-seeking behaviors; hence, culturally sensitive, tailor-made counseling and awareness interventions should be provided.

The findings also show that one-third of the respondents lack awareness about contraceptive methods. Especially, it is very significant for psychiatric outpatient care. Many patients are not able to access contraceptive-related information, which may contribute to unmet contraceptive-related information needs and unwanted pregnancies, and reduced autonomy in reproductive decision-making. Reproductive choices are very important and a serious concern for persons with mental illness. After marriage, mentally ill persons are not allowed to make decisions regarding their reproductive choices. Women with mental illness face systematic challenges due to the preconceived idea that women with mental illness cannot make decisions on their own. Hence, they are forced into sterilization, denial of reproductive agency, and more likely to experience infringement of their sexual and reproductive rights, as well as human dignity.

Exercise of liberty and freedom of choice in reproduction

The current study findings show that 37.45% of respondents had planned to have a child, which is similar to other studies [37,38]. However, in only in 49.95% cases, respondents were the sole decision-makers to have a child (43.4% (36) of women with schizophrenia and 37.3% (25) of women with BPAD), and 80.7% of women were not involved in the decision to have a child. This is in line with the study conducted by Gupta et al. [39]. The present study finding reflects that 42.9% of respondents made independent decisions related to marriage. Another study supports the present findings, which indicate that persons with BPAD tend to maintain greater family relationships and make decisions on their own than persons with schizophrenia [40]. This is in line with the study conducted by Khatoon et al. [41]. The study findings indicate that 24.95% of respondents have never heard about family planning methods. Various research studies conducted across the country report a lack of adequate knowledge regarding various family planning methods among women [42,43]. This may be attributed to the socio-cultural factors, including restrictions imposed by the family on females, inadequate dissemination of information on family planning methods, religious beliefs, stigma, and attitudes towards contraceptives. The current study also found that a very small number of participants reported being sexually controlled. This may not be interpreted as there is no control by the partners, keeping the socio-cultural arena of the Indian society, where the words of women are often suppressed, unheard, or underrepresented. It is crucial that, though there may be chances of sexual control by the partners, this may not be revealed openly by the women. Hence, there may be a chance of potential underreporting or discomfort with disclosing coercion.

This is a cross-sectional study set in a tertiary psychiatric care facility in an urban setting. The group was predominantly diagnosed with schizophrenia and BPAD. However, this study offers considerable insights that women with mental illnesses are deprived of their reproductive rights. Thus, the government should focus on the well-being of women with mental illness, promoting dignity and quality of life. Furthermore, there should be adequate healthcare facilities where people can access them easily without barriers or discrimination. The data collection was conducted between August 2023 and May 2024. During this period, a few challenges were encountered with data collection, including obtaining timely ethical clearance from the institute; difficulties in recruiting participants, as many participants were reluctant to discuss the sensitive reproductive health issues; and symptom-related difficulties, making them uneasy during the interview. Additionally, conducting interviews with multilingual participants and participants coming from a long distance were the difficulties we faced during the recruitment of samples. 

Limitations of the study

The limitations of the study were employing a cross-sectional design rather than a longitudinal study, and it does not establishes causal relationship as it is descriptive in nature. The study was conducted in a single centre, an urban-based psychiatric hospital, which may limit the establishment of significant associations. Therefore, it may not be an accurate representation of women with schizophrenia and BPAD.

## Conclusions

The present study examined the knowledge and awareness of reproductive rights among women with severe mental illness in an urban population in India. The findings demonstrate that some participants lack understanding of basic knowledge about reproductive health issues and need more information about reproductive rights. The findings suggest limited autonomy in selected reproductive decisions, especially concerning marriage, childbearing, and family planning, highlighting that constraints are context-specific rather than uniformly experienced across all aspects of reproductive choice. Promoting reproductive rights is essential and a need for us in India. Therefore, all stakeholders, such as government institutions and non-governmental organizations, community leaders, and healthcare systems, should come forward to address this issue. Enhancing awareness and decision-making capacity is essential to ensure dignity, equality, and human rights for this vulnerable population. The findings show limited autonomy in selected reproductive decisions, especially concerning marriage, childbearing, and family planning, highlighting that constraints are context-specific rather than uniformly experienced across all aspects of reproductive choice.

## Appendices

Table 4 presents the questionnaire for knowledge and awareness of reproductive rights among women with severe mental illness.

**Table 4:** Questionnaire for knowledge and awareness of reproductive rights among women with severe mental illness

Sl. No	Questions	Yes/No
Awareness about the fundamental rights		
1	Are reproductive rights fundamental rights?	
2	Apart from your husband, have you had any other sexual partners in the last 12 months?	
3	Do you feel restrictions imposed on girls during menstruation is right move?	
Knowledge and awareness about reproductive health		
4	Do you feel that unjustified medication can harm you and your reproductive health?	
5	Do you feel medication should be provided to meet the best health needs of the patient?	
5	Do you feel sterilization should not be carried out as a treatment for mental illness?	
7	Are you aware that during an illness period, conceiving or giving birth could impact your mental health?	
8	Do you have knowledge of how to prevent unwanted pregnancy?	
9	The major source of information regarding puberty and menstruation?	
10	The source of information regarding menstruation before reaching the age of menarche?	
11	Are you aware of the different contraceptive methods available in India?	
12	Do you know about injectables as a type of contraceptive?	
13	What is the source of information on contraceptives?	
14	Is it healthy to get your period at a young age?	
15	Do you feel girls should be provided with proper education and awareness to help them become emotionally and physically ready?	
16	Do you feel girls should be educated in maintaining the highest level of hygiene during their periods?	
17	Do you ever feel lack of access to high-quality hygiene products is a major impediment?	
18	Do you agree that women and girls do not have access to adequate sanitation facilities?	
19	Do you feel girls should receive comprehensive counselling on menstruation and other associated issues?	
Exercise of liberty and freedom of choice in reproduction		
20	Have you ever planned to have a child?	
21	Have you ever had a miscarriage or aborted or stillbirth child?	
22	Has your partner ever controlled you sexually?	
Women’s Autonomy in reproductive health decision making		
23	Did you feel you should have a baby now or later?	
24	Are you solely take decision to have a child?	
25	Was your marriage being solemnized on your own choice?	
26	Would you plan to have a child in the near future?	
27	Do you ever hear about family planning methods?	
